# The effect of multiple blood-feeding on the longevity and insecticide resistant phenotype in the major malaria vector *Anopheles arabiensis* (Diptera: Culicidae)

**DOI:** 10.1186/1756-3305-7-390

**Published:** 2014-08-23

**Authors:** Shüné V Oliver, Basil D Brooke

**Affiliations:** Centre for Opportunistic, Tropical and Hospital Infections, National Institute for Communicable Diseases/NHLS, Sandringham, Johannesburg, South Africa; Wits Research Institute for Malaria, School of Pathology, Faculty of Health Sciences, University of the Witwatersrand, Johannesburg, South Africa

**Keywords:** Blood meal, Insecticide resistance, Oxidative stress

## Abstract

**Background:**

*Anopheles arabiensis* is a major malaria vector in Africa. Adult females are likely to imbibe multiple blood meals during their lifetime. This results in regular exposure to potential toxins and blood-meal induced oxidative stress. Defence responses to these stressors may affect other factors of epidemiological significance, such as insecticide resistance and longevity. The aims of this study were to examine the effect of multiple blood-feeding on insecticide tolerance/resistance with increasing age, to assess the underlying biochemical mechanisms for the responses recorded, and to assess the effect of multiple blood-feeding on the life histories of adult females drawn from insecticide resistant and susceptible laboratory reared *An. arabiensis*.

**Methods:**

Laboratory reared *An. arabiensis* females from an insecticide resistant and an insecticide susceptible colony were offered either a single blood meal or multiple blood meals at 3-day intervals. Their tolerance or resistance to insecticide was then monitored by WHO bioassay four hours post blood-feeding. The biochemical basis of the phenotypic response was assessed by examining the effect of blood on detoxification enzyme activity and the effect of blood-meals on detoxification enzyme activity in ageing mosquitoes.

**Results:**

Control cohorts that were not offered any blood meals showed steadily decreasing levels of insecticide tolerance/resistance with age, whereas a single blood meal significantly increased tolerance/resistance primarily at the age of three days. The expression of resistance/tolerance in those cohorts fed multiple blood meals generally showed the least variation with age. These results were consistent following exposure to DDT and pyrethroids but not to malathion. Multiple blood-meals also maintained the DDT and permethrin resistant phenotype, even after treatment females had stopped taking blood-meals. Biochemical analysis suggests that this phenotypic effect in resistant females may be mediated by the maintenance of increased glutathione s-transferase activity as a consequence of multiple blood-feeding. Multiple blood-feeding increased the longevity of insecticide resistant females regardless of their mating status, but only increased the longevity of unmated susceptible females.

**Conclusion:**

These data suggest that multiple blood-feeding confers a competitive advantage to insecticide resistant females by increased longevity and maintenance of the expression of resistance with age.

## Background

*Anopheles arabiensis* is a major malaria vector in Southern and Eastern Africa. It is a member of the *An. gambiae* species complex and often occurs in sympatry with the major malaria vectors *An. gambiae sensu stricto, An. coluzzii* and *An. funestus* [1] *.* Although it is not as efficient a vector as *An. funestus* and *An. gambiae* [1], *An. arabiensis* does present a unique challenge to vector control efforts because it is endophilic and exophilic and contributes significantly to outdoor malaria transmission where it occurs [2]. Exophilic *An. arabiensis* are not susceptible to control by indoor residual spraying and are not well controlled by conventional and long-lasting insecticide treated nets either [3].

Haematophagy in anautogenous mosquitoes, including *An. arabiensis*, has clear reproductive functions. Blood-feeding, however, has various biological consequences not related to egg production. Blood-feeding sets off a variety of biological processes, with many transcriptional changes peaking at 3 hours after feeding [4]. These changes have an impact on characteristics that affect vector control. Previous studies on various mosquito species have demonstrated that insecticide toxicity is reduced after a single blood-meal [5–9]. A disease vector mosquito female requires two meals to be of epidemiological significance and it is highly likely that during her lifetime a female will take multiple blood meals [10]. Therefore, a question arises as to what effects multiple blood-feeding has on ageing mosquitoes.

In terms of malaria epidemiology and vector control there are two important considerations. The first is the effect of blood-feeding on longevity. Small changes in longevity have profound consequences on disease transmission [11]. A combination of blood and sugar as a dietary combination has been shown to provide the best survival in *An. gambiae* [12]. It is important, however, to know whether this holds equally true for insecticide resistant and susceptible populations.

It is also important to determine the effect of multiple blood-feeding on the expression of insecticide resistance in senescent mosquitoes. It is well established that sensitivity to insecticides increases with age [10–16]. This is assumed to be a function of decreasing enzyme activity due to the reduction of soluble proteins with age, particularly in insecticide resistant mosquitoes [14–19]. It has been suggested that the effect of age is more important in the expression of insecticide resistance than larval nutrition [20], and that a recorded decline in DDT resistance with age in *An. gambiae* from Zanzibar was not an effect of undernourishment, but also occurred in natural populations taking regular blood-meals [16]. Furthermore, several studies have demonstrated that blood-feeding does not influence the decline of insecticide resistance in older mosquitoes [21–23]. This has led to the suggestion that ageing may partially restore the efficacy of insecticide based vector control [24].

The aims of this study were to examine the effect of multiple blood-feeding on insecticide tolerance/resistance with increasing age, to assess the underlying biochemical mechanisms for the responses recorded, and to assess the effect of multiple blood-feeding on the life histories of adult females drawn from insecticide resistant and susceptible laboratory reared *An. arabiensis*.

## Methods

This study examined the effect of multiple blood-meals on the resistant phenotype up to the age of 21 days. By this age the female will have passed her extrinsic incubation period [25] and will be of epidemiological significance as a potential malaria vector. The underlying biochemical mechanisms for these responses were also examined.

### Laboratory strains

Two laboratory strains of *An. arabiensis* were used in these experiments. SENN is an insecticide susceptible strain from Sennar, Sudan. SENN-DDT is a strain selected for DDT resistance from the SENN strain. The SENN-DDT strain was selected by 1 hour exposures of 1 – 3 day old adult mosquitoes to 4% DDT, and survivors were allowed to breed. This procedure was followed through 100 generations prior to these experiments. This selected strain is resistant to DDT, permethrin, deltamethrin and malathion (unpublished data). Resistance is mediated by elevated cytochrome P450, glutathione S-transferase (GST) and general esterase activity. The mosquitoes are also fixed the L1014F *kdr* mutation [26]. All mosquitoes were reared as described by Hunt *et al.* [23].

### Experimental set-up

Adult mosquitoes were subjected to one of three dietary regimens. The control groups were not offered blood throughout their lives, and were maintained only on 10% sucrose solution. This constituted the sugar fed control treatment. The second group was maintained on sucrose solution, but were allowed a single blood-meal at the age of 3, 7, 11, 15, 18 or 21 days. This constituted the single blood-meal treatment group. The third group was maintained by feeding on sucrose solution, but allowed blood at the age of 3, 7, 11, 15, 18 and 21 days. At each time point, a sub-section of the population was removed for bioassays. Therefore, the population removed at 7 days of age had 2 blood-meals; the population removed at 11 days had 3 blood-meals etc. This group constituted the multiple blood-fed treatment group. In this study a cohort is defined as a sample drawn from a single egg batch. For each treatment, 3 cohorts were used, and for every age group, three replicates (individual exposures) were performed. Mosquitoes that were bioassayed were not returned to their respective populations.

Samples were drawn from each dietary treatment at the ages of 3, 7, 11, 15, 18 and 21 days post emergence. Those that were allowed blood in their diet were fed to repletion. Four hours after feeding, individuals were either exposed to insecticide using the WHO bioassay or kept for biochemical analysis. Those kept for biochemical analysis were aged for a further 72 hours to allow those that had taken one or more blood-meals time to digest the blood completely.

### Insecticide susceptibility assays subsequent to blood-feeding

For blood-feeding treatments, females were allowed to feed to repletion on the arm of a human volunteer. All procedures performed were approved by the human ethics committee of the University of the Witwatersrand (Clearance certificate M130534). A single, consenting human volunteer was used as a previous study demonstrated that the blood of a human augmented pyrethroid resistance better than that of an anaesthetised guinea pig. Furthermore, the use of a single human volunteer has not been shown to have any effects uniquely related to the volunteer [27]. Throughout their lives, males and females were kept together and allowed to mate. Females were allowed to oviposit twice a week for the duration of the experiment.

Insecticide susceptibility assays (bioassays) were performed on samples drawn from each treatment cohort within the insecticide resistant SENN-DDT strain at each age interval. In the blood-fed cohorts, females were allowed to feed to repletion. Four hours after the meal, samples from all three dietary treatments were exposed to test papers treated with either 4% DDT, 0.75% permethrin, 0.05% deltamethrin or 5% malathion for 1 hour, and mortality was scored 24 hours post exposure according to the standard WHO procedure [28]. Sample sizes are summarised in Table 1. All insecticide treated papers were obtained from the School of Biological Sciences, Universiti Sains Malaysia (USM), Penang, Malaysia, After exposure, females were allowed *ad libitum* access to 10% sucrose. Bioassay controls included samples exposed to untreated papers. Final mortalities 24 h post exposure was compared between treatments by age.

**Table 1:** **Sample sizes of** ***Anopheles arabiensis*** **(SENN-DDT) insecticide treatments by age**

Treatment	Age	DDT	Permethrin	Deltamethrin	Malathion
**Sugar fed**	3 days	714	696	695	813
	7 days	476	464	463	543
	11 days	476	464	463	542
	15 days	476	464	463	-
	18 days	476	464	463	-
	21 days	476	464	463	-
**Single blood meal**	3 days	714	696	695	813
	7 days	476	464	463	543
	11 days	476	464	463	542
	15 days	476	464	463	-
	18 days	476	464	463	-
	21 days	476	464	463	-
**Multiple blood meals**	7 days	476	465	463	543
	11 days	476	464	463	543
	15 days	476	464	463	-
	18 days	476	464	463	-
	21 days	476	465	463	-
**Total:**		**8568**	**8354**	**8335**	**4882**

### Delayed blood-feeding bioassays

The aim of this experiment was to determine whether multiple blood-meals had a lasting effect on the insecticide resistant phenotype even after the meals were halted. For this procedure, the three dietary treatment regimes of the previous experiment were followed. However, at day 15, the multiple blood-meal treatment was split into two groups. One group was not offered any more blood, and the second group was given another blood-meal at the age of 18 days. At 18 days, the sugar fed group, the single blood meal group, the multiple blood-meal group that had meals up to the age of 18 days and the multiple blood-meal group that had meals up to the age of 15 days (the delayed exposure group) were exposed by WHO bioassays as described above. Final mortalities 24 h post exposure was compared between treatments as well as between the two multiple blood-meal groups.

### Biochemical analysis

Insecticide resistance by increased metabolic detoxification is mediated by one or a combination of three enzyme superfamilies; the cytochrome P450s, Glutathione S-transferases (GSTs) and general esterases. Analysis of these enzyme activities give an indication of the mechanism underlying the insecticide resistance [29, 30] Recently, ABC transporters have been implicated in insecticide resistance [31, 32] but have not been implicated in insecticide resistance in the laboratory strains of *An. arabiensis* used in this study. Previous analysis of the mechanisms underlying the insecticide resistance phenotype in the SENN-DDT strain has been attributed to the *kdr* mutation as well as elevated activities of α- and β-esterases, ctyochrome P450s and GSTs [26]. Therefore, the effect of blood-feeding was examined on these particular enzyme systems.

Biochemical analysis is normally performed on mosquitoes that have not had a blood-meal, as per WHO guidelines [28]. This is because host components of the blood-meal could interfere with the analysis, as the ingested proteins interact with the assay substrates [28]. This study, however, aimed to determine the effect of blood-feeding treatment on the activity of detoxification enzymes with time. To avoid host protein substrate interference, when samples from the different nutritional regimens were assayed for detoxification enzyme activity, specimens were killed 72 hours after their last feeding treatment. This had the two-fold effect of allowing complete digestion of the blood-meal, and also enabled analysis of the lasting effect of the dietary treatment in each individual. Therefore, in this experiment, although the same time points were used as for the bioassays, chronologically the mosquitoes were 3 days older. For example, in the single point detoxification analysis described below the mosquitoes examined are labelled as 15 days old, but when assayed they were 18 days old. This is because the labelling system reflects their meal regimen rather than their age. For detoxification assays, all specimens were homogenised in PCR grade water after being killed at -70°C.

### Single point biochemical assays

As DDT and pyrethroid resistance were most strongly affected by multiple blood-meals, the cytochrome P450 and GSTs were analysed for females drawn from the three treatment regimens that were allowed their last meal or only blood-meal at the age of 15 days. Cytochrome P450 activity was determined as a function of haeme peroxidase activity [29]. GST activity was determined as 1-Chloro-2,4-dinotrobenzene (CDNB) conjugation activity [33]. Proteins were quantified using the Bradford method [30]. The GST and cytochrome P450 activities of samples from SENN and SENN-DDT were determined. Enzyme activities between strains by treatment and between treatments within each strain were compared. For all detoxification enzyme analysis 96 individuals were used for age and blood feeding regimen for each strain.

### Biochemical analysis of ageing adult mosquitoes

Previous studies have demonstrated that a single blood-meal enhances the expression of pyrethroid resistance in pyrethroid resistant females [9]. As the previous experiment also demonstrated that blood-feeding affected the expression of resistance in the insecticide resistant SENN-DDT strain, it was decided to analyse the effect of blood-feeding on enzyme activity with age in SENN-DDT. As previously described, samples of 50 females drawn from each dietary treatment at each successive age were killed 72 hours after treatment. Males from each age cohort were also killed, and their enzyme activities used for comparison. Proteins were quantified and Cytochrome P450 and GST activities were determined as previously described. Additionally, general esterase activity was also determined [30]. Enzyme activities by age were compared between treatments and genders. For all detoxification enzyme analysis 96 individuals were used for age and blood feeding regimen for each strain.

### Longevity

To determine the effect of multiple blood-feeding on the longevity of SENN and SENN-DDT females, newly emerged males and females were separated. Samples of unmated females provided with 10% sucrose solution only served as a control. 30 unmated females were used as the unmated blood-fed cohort. 30 mated females (placed in a cage with 45 males and allowed to mate for the duration of the experiment) served as the mated blood-fed cohort. The mated and unmated cohorts were blood-fed to repletion twice weekly and a moistened filter paper was provided twice weekly to allow for oviposition. To determine whether any effects observed were due to blood-feeding or due to additional protein supplementation, 30 unmated females were provided with 10% sucrose supplemented with 50 mM Bovine Serum Albumen (BSA). These females were not offered any blood-meals. Longevity of each treatment cohort was monitored until all females had died. For each treatment, three technical repeats were performed with three different cohorts, resulting in a total of nine replicates per treatment. Mean longevity was compared between treatment cohorts.

### Statistical analysis

All statistical analyses were performed using Statistix 8 (Tallahassee, Fl, USA) and Statistica 10 (Statsoft, USA). All means were compared using either a 2-sample t-test or a 1-way Analysis of Variance (ANOVA) with a Tukey comparison-of-means as a post-hoc test. All confidence intervals were set at 95%. Abbott’s formula was not applied to bioassay data as control mortalities did not exceed 10% at any point. Longevity was assessed using the Kaplan-Meier estimator, using the log-rank test to determine the significance as none of the data were censored.

## Results

### Bioassays

**Figure 1:**
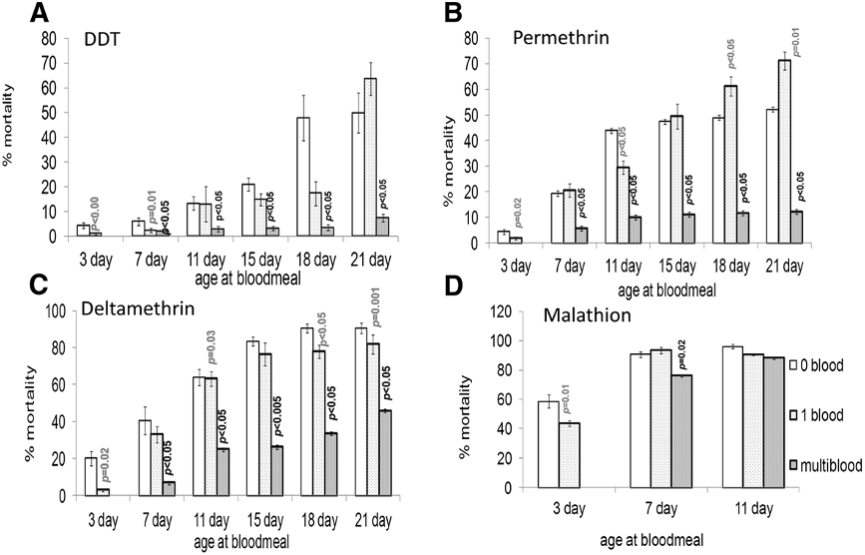
**The effect of blood feeding on insecticide induced mortality in** ***Anopheles arabiensis*** **SENN-DDT females.** Mean percentage mortalities 24 hours post exposure are given for DDT **(A)**, permethrin **(B)**, deltamethrin **(C)** and malathion **(D)**. Insecticide bioassays were conducted 4 hours post blood meal and mortalities are plotted by age and blood feeding regimen i.e. sugar fed only (0 blood), single blood meal (1 blood), multiple blood meals (multiblood). P-values obtained by 2-sample test indicate degree of significance in differences in mortalities between blood-fed cohorts and their respective sugar fed control cohorts.

For all insecticides tested, a single blood meal at the age of three days significantly reduced insecticide-induced mortality relative to sugar-fed females (Figure 1A, B, C and D). Malathion bioassays were not performed after mosquitoes were 11 days old. Mortality for all treatments exceeded 90% at that age, and, although a single blood meal at 3 days and multiple blood meals by 7 days significantly reduced mortality, blood feeding appeared to cease having an effect on susceptibility by then (Figure 1D). For DDT, permethrin and deltamethrin treatments, multiple blood-meals always resulted in significantly lower levels of mortality compared to sugar fed control females of the same age (Figure 1A, B, C). For mosquitoes exposed to DDT, the mortalities of cohorts fed a single meal at the age of 3 days and those fed multiple blood meals did not differ significantly between the ages of 3 to 18 days (1-Way ANOVA: p = 0.36; F = 1.11 df = 1) (Figure 1A). For permethrin-exposed females, there was no significant variation in mortality in the multiple-fed cohort between the ages of 7–21 days (1-Way ANOVA: p = 0.35; F = 1.13, df = 1) (Figure 1B).

Single blood meals induced a variable effect on mortality, only consistently showing significantly reduced mortality compared to controls in 3 day old mosquitoes (Figure 1A, B, and C, D). Notable exceptions to this single blood meal trend are the significant increase in permethrin-induced mortality at 18 and 21 days (1-Way ANOVA: p < 0.05 F = 6.35; df = 1) (Figure 1B), and the significantly reduced deltamethrin mortality at the age of 18 and 21 days (1-Way ANOVA: p < 0.05; F = 4.98; df = 1) (Figure 1C).

**Figure 2:**
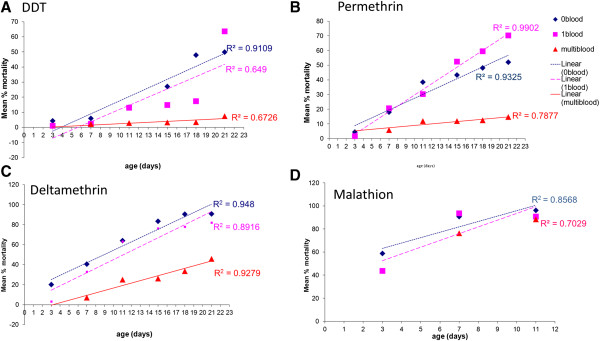
**Linear regression analysis of insecticide-induced mortality by age by blood feeding regimen (sugar fed only - 0 blood, single blood meal - 1 blood, multiple blood meals – multiblood) in** ***Anopheles arabiensis*** **SENN-DDT females.** Insecticide bioassays were conducted 4 hours post blood meal at each age interval indicated and mean percentage mortalities 24 hours post exposure are shown for DDT **(A)**, permethrin **(B)**, deltamethrin **(C)** and malathion **(D)**. Goodness-of-fit for each regression is indicated by R^2^ values. No R^2^ value is given for multiple-feeding for the malathion treatment as only two points were available for analysis.

As mosquitoes age, they are more likely to die when exposed to insecticide. In Figure 2, a strong linear relationship between mortality and age is apparent, particularly in sugar-fed individuals exposed to DDT (Pearson’s correlation r = 0.9; p < 0.05); permethrin (Pearson’s correlation r = 0.9; p < 0.05) and deltamethrin (Pearson’s correlation r = 0.9; p < 0.05). When females were provided with a single blood-meal, there was still a strong linear relationship between age and mortality for permethrin treated females (Pearson’s correlation r = 0.9; p < 0.05) (Figure 2B) and deltamethrin (Pearson’s correlation r = 0.9; p < 0.05) (Figure 2C), but a weaker relationship for those exposed to DDT (Pearson’s correlation r = 0.6; p = 0.05) (Figure 2A). For individuals fed multiple meals, there was a comparatively weak linear relationship between age and mortality for the DDT exposed cohorts (Pearson’s correlation r = 0.6; p = 0.08), whilst for deltamethrin treated cohorts, mortality increased in a linear fashion with age (Pearson’s correlation r = 0.9; p < 0.05). In Table 2 it is apparent that for DDT, permethrin and deltamethrin treated individuals mortality of the single blood-meal treated individuals did not vary with age compared to those cohorts that were not offered blood, but the mortalities of those that had multiple blood-meals were significantly lower. Statistical indicators are summarised in Table 2.

**Figure 3:**
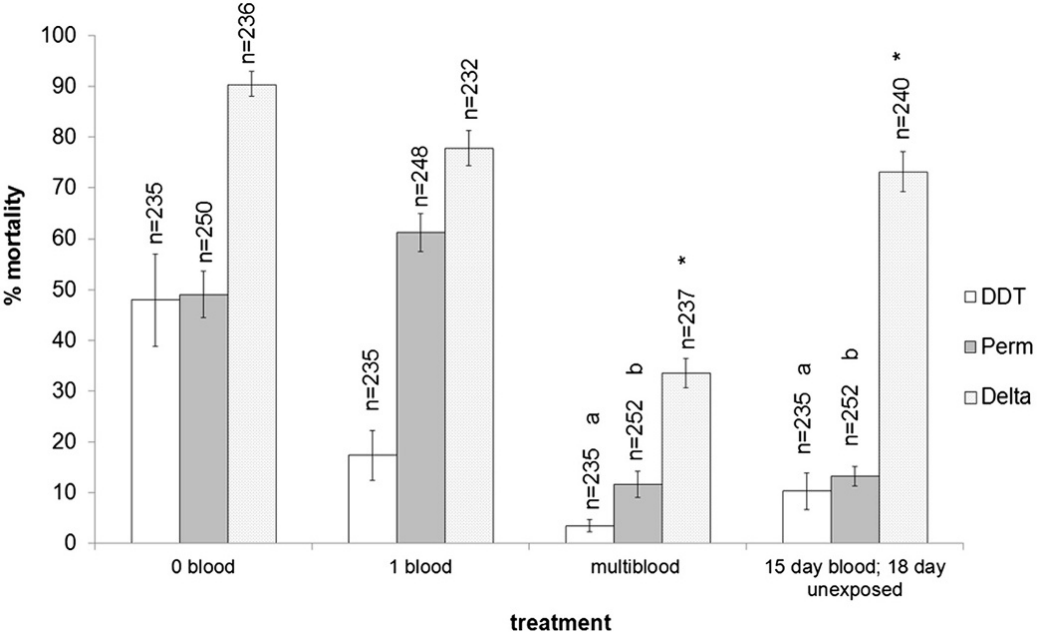
**The lasting effects of multiple blood-feeding on the expression of insecticide resistance in** ***Anopheles arabiensis*** **SENN-DDT females.** Mean percentage mortalities of 18 day old SENN-DDT females that either had no blood, a single blood meal (1 blood) or their fifth blood meal (multiblood) 4 hours prior to exposure were compared to that of 18 day old females that acquired their last blood meal at the age of 15 days (15 day blood; 18 day exposed). Insecticide bioassays were conducted 4 hours post blood meal except for the last cohort which was assayed against listed insecticides 3 days post blood-feeding (15 day blood; 18 day exposed). Only the mean mortality induced by the delayed deltamethrin treatment differs significantly from the mean mortality of the multiple blood meal cohort (multiblood) of the same age (p < 0.05). The effect of multiple blood-feeding on the expression of resistance was maintained for the DDT and permethrin resistant phenotypes 3 days post blood-feeding. Perm = permethrin; Delta = deltamethrin. The asterisks denote significant differences between 18 day multiple blood meal treatments and those exposed at 18 days but last fed 3 days prior to exposure. Lower case letters indicate no significant differences.

Following experiments to determine whether multiple blood meals had an effect on mortality in the long term or only after each meal, it was shown that mortality in the delayed exposure group did not differ significantly from those fed multiple meals in the cohorts exposed to either DDT (2 sample t-test: p = 0.10; t = -1.80) or permethrin (2 sample t-test: p = 0.88; t = -0.15), but was significantly higher in cohorts exposed to deltamethrin (2 sample t-test: p < 0.05; t = -8.94) (Figure 3).

**Table 2:** **One way ANOVA comparing insecticide-induced mortality of unfed** ***Anopheles arabiensis*** **SENN DDT females at different ages to their counterparts fed either a single or multiple blood meals**

	DDT	Permethrin	Deltamethrin	Malathion
1 blood	p = 0.64 (F = 0.23;df = 1)	p = 0.83 (F = 0.05;df = 1)	p = 0.61 (F = 0.26;df = 1)	p = 0.78 (F = 0.09;df = 1)
Multiblood	p = 0.04 (F = 5.16;df = 1)	p = 0.02 (F = 8.56;df = 1)	p = 0.039 (F = 6.76;df = 1)	p = 0.98 (F = 0.00;df = 1)

### Detoxification enzyme analysis

**Figure 4:**
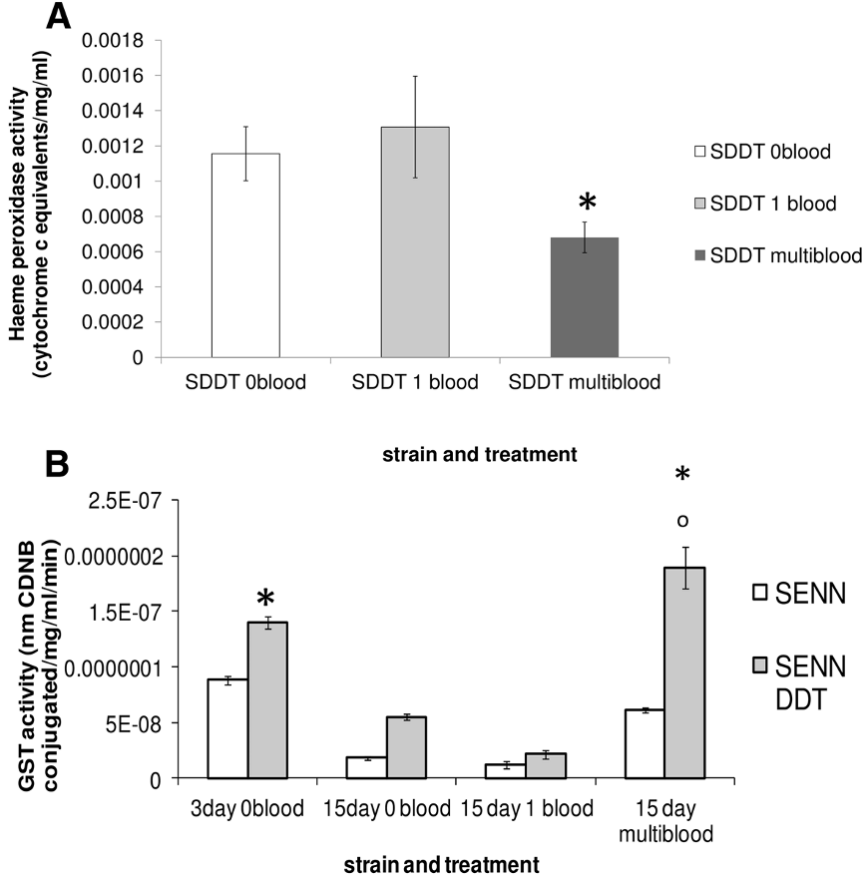
**Comparison of detoxification enzyme activity of 18 day old SENN and SENN-DDT** ***Anopheles arabiensis*** **females.** 15 day old females either deprived of blood (15 day 0 blood), offered only one blood meal at 15 days (15 day 1 blood) or fed multiple blood meals at three day intervals up to and including 15 days (15 day multiblood) were allowed 72 hours to digest their final blood meal (if offered) before being assayed for detoxification enzyme activity. **Block A** represents haeme peroxidase activity and shows a comparison of SENN-DDT females that had no blood meals compared to those that had single or multiple meals. The asterisk denotes a significantly lower enzyme activity in multiple blood-fed SENN-DDT compared to all other cohorts. SENN is not represented as there were no significant changes in haeme peroxidise activity in this strain regardless of dietary treatment. **Block B** represents Glutathione S-transferase (GST) activity of SENN and SENN-DDT females. The asterisks denote a significant difference between GST activities of the different strains at the same age. A circle denotes a significant increase in activity of a 15 day old SENN-DDT cohort compared to their 3 day old counterparts.

The cytochrome P450 and GST activity of 15 day old females allowed 72 hours to digest their blood meal (age at analysis is therefore 18 days) were analysed to determine how the effects of multiple blood feeding on the resistance phenotypes are mediated. Cytochrome P450s and GSTs were analysed in particular as the multiple blood meals had a sustained effect on pyrethroid and DDT resistance. Haeme peroxidase activity, a measure of Cytochrome P450 activity, was significantly reduced in SENN-DDT females that had multiple blood meals (2-sample t test: p = 0.01; t = 2.75) (Figure 4A). GST activity in SENN-DDT females that had multiple blood meals was significantly higher than females of the same strain and age that had not been offered blood (2-sample t-test: p < 0.05, t = -7.74), as well as females that had taken a single blood meal (2-sample t-test: p < 0.05, t = -11.88). GST activity in SENN-DDT females that had multiple blood meals was significantly higher than SENN females that had no blood (2-sample t-test: p < 0.05, t = 9.22), a single blood meal (2-sample t-test: p < 0.05, t = 9.44) or multiple blood meals (2-sample t-test: p < 0.05, t = 6.86). When compared to unfed 3-day old SENN-DDT females, 18-day old SENN-DDT females that had taken multiple meals showed significantly higher levels of GST activity (2 sample t-test p = 0.01; t = -2.59) (Figure 4B). In contrast, although multiple blood meals significantly increased GST activity in SENN females compared to those of the same age that had no blood meals (2 sample t-test: p < 0.05; t = -17.31) or a single meal (2 sample t-test: p < 0.05; t = -12.54), the GST activity of unfed 3 day old females had significantly higher levels of GST activity than those that had multiple blood meals (2 sample t-test: p < 0.05; t = -5.99).

**Figure 5:**
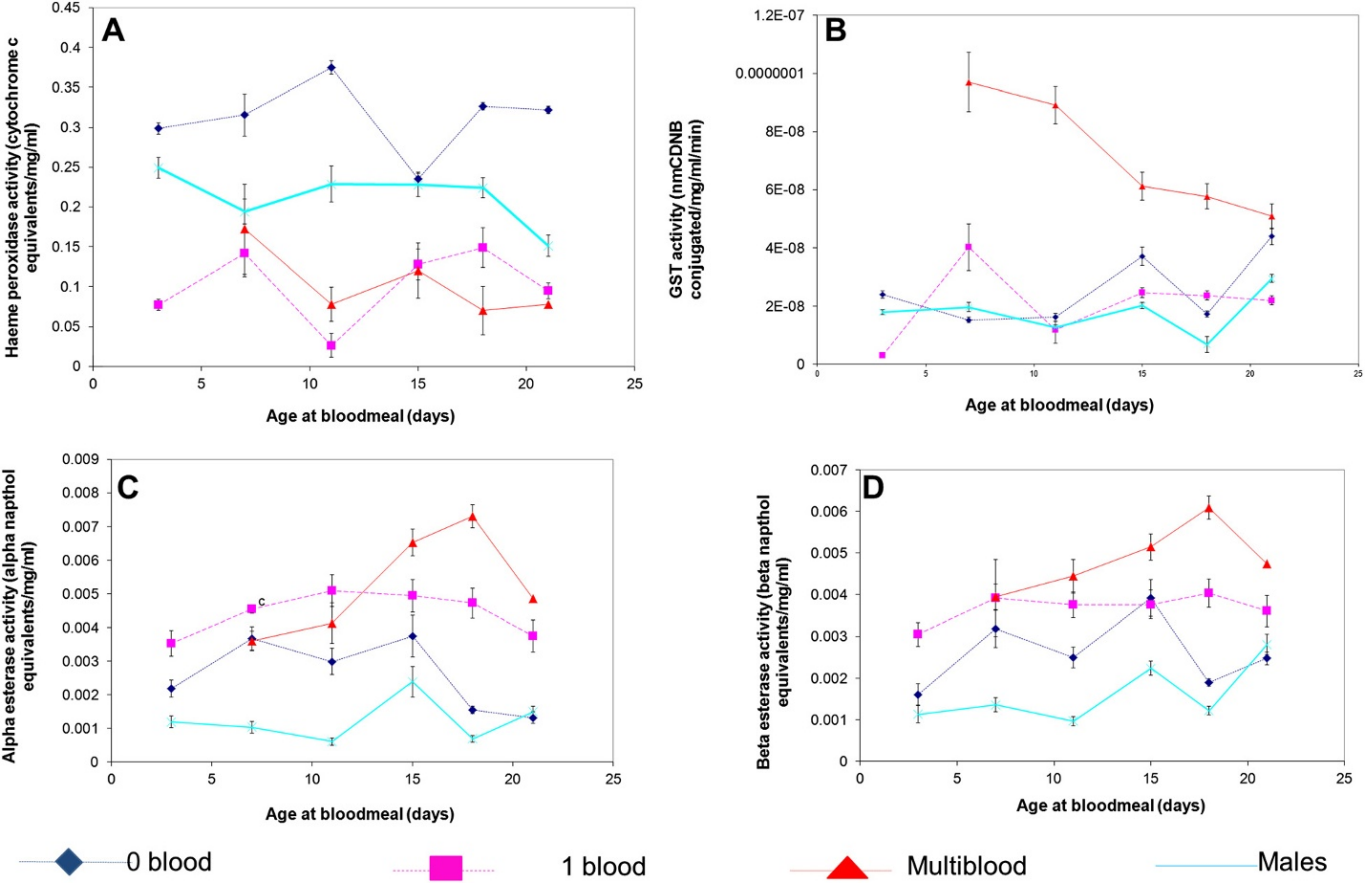
**Detoxification enzyme activities of** ***Anopheles arabiensis*** **SENN-DDT by age and as a function of blood feeding regimen in females. Block A** represents Cytochrome P450 activity as measured by haeme peroxidase activity, **Block B** represents GST activity, **Block C** represents α esterase activity and **Block D** β esterase activity. Enzyme activities in males were measured as baseline levels against which the enzyme activities of unfed, single blood meal and multiple blood meal cohorts of females were compared.

Multiple blood meals exerted a significant effect on the detoxification enzyme activity of insecticide resistant, but not susceptible females. An analysis of detoxification enzyme activity over time as affected by blood meals was therefore conducted on SENN-DDT females only, with males of the same age used as a comparison (Figure 5). The same age intervals were used as for the bioassays, with 72 hours allowed for blood digestion. Therefore, although the ages of 3 until 21 days are indicated, analysis actually took place 3 days afterwards. The Cytochrome P450 activity of non blood-fed females was always significantly higher than those that had either a single or multiple meals (1-way ANOVA p < 0.05; F = 37.9; df = 2) (Figure 5A). Females that had blood meals, either single or multiple, always had significantly lower levels of Cytochrome P450 activity than their male counterparts (1-way ANOVA p < 0.05; F = 13.9; df = 3). Only at the age of 18 days (1-way ANOVA p < 0.05; F = 27; df = 1) do the single and multiple blood meal cohorts differ significantly from each other. The GST activity (Figure 5B) of females that had multiple blood meals declined steadily throughout their lives, but always remained significantly higher than those of males or females that had one or no blood meals (1-way ANOVA p < 0.05; F = 37.9; df = 3). A single blood meal at the age of 3 days significantly increased both α- and β-esterase activity (2 sample t-test: p < 0.05; t = 5.63 for α-esterases and p < 0.05; t = -4.48 for β-esterases). Multiple blood meals significantly increased α- and β-esterase activity from the age of 15 days compared to the no blood, single blood and male cohort (1-way ANOVA: p < 0.05; F = 20.4; df = 3 for α-esterases and p < 0.05; F = 15.8; df = 3 for β-esterases) (Figure 5C; Figure 5D).

### Longevity

**Figure 6:**
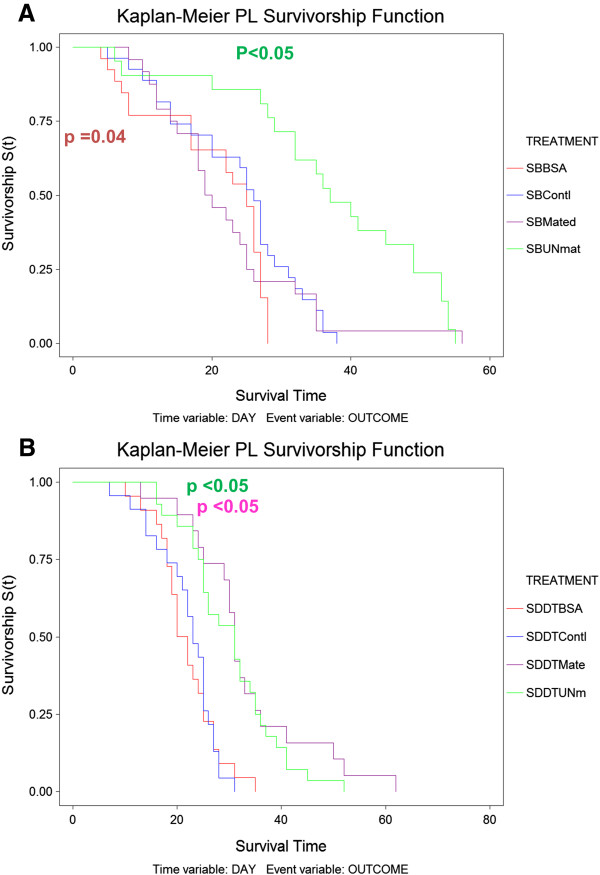
**The effect of multiple blood-feeding on the longevity (survivorship) of** ***Anopheles arabiensis*** **SENN and SENN-DDT females (Blocks A and B respectively).** Light blue and pink lines represent unmated and mated respectively. The effect of Bovine Serum Albumin (BSA) on the longevity of SENN and SENN-DDT females are also displayed, with the BSA treated females represented by the dark blue line. The longevity of all the treatments were compared to that of the sugar fed controls (red line).

Blood-feeding had a differing response on resistant and susceptible strains. BSA was used as a control to determine whether the effects induced by blood meals were due to blood itself, or simply additional dietary protein. BSA significantly reduced the longevity of SENN females compared to those that only had a sugar meal (Log rank test: p = 0.04; χ^2^ = 8.68) (Figure 6A), but not SENN-DDT females (Log rank test: p = 0.92; χ^2^ = 0.01) (Figure 6B). Multiple blood meals significantly increased the longevity of mated (Log rank test: p < 0.05; χ^2^ = 13.74) and unmated SENN-DDT females (Log rank test: p < 0.05; χ^2^ = 11.21) (Figure 6B). For SENN females, multiple blood meals increased longevity in unmated females (Log rank test: p < 0.05, χ^2^ = 5.29), but not mated females (Log rank test: p = 0.14, χ^2^ = 2.22), (Figure 6A).

## Discussion

Blood feeding stimulates a variety of metabolic processes in female mosquitoes, and several blood meals may be taken during a gonotrophic cycle, which may result in the triggered changes occurring in a cyclical pattern [34]. This response is important for the gonotrophic cycle and is likely to affect xenobiotic detoxification as well. This is because blood feeding induces changes in the regulation of detoxification enzymes so as to enable the female to cope with the metabolic challenge posed by blood ingestion [34]. Previous studies have assessed the effect of a single blood meal on the expression of insecticide resistance [8, 9], but these data are the first to demonstrate the maintenance of the expression of insecticide resistance as a consequence of multiple blood-feeding.

The *An. arabiensis* insecticide resistant strain used in this experiment, SENN-DDT, mediates resistance by a combination of *kdr* and metabolic mechanisms [26]. In the absence of blood feeding, the expressions of DDT, pyrethroid and malathion resistance decline with age in SENN-DDT despite the presence of *kdr*. However, multiple blood-feeding either arrests or reduces this decline with age. Generally, *kdr* is most closely associated with DDT resistance, followed by Type I pyrethroids, followed by Type II pyrethroids [35, 36]. In the SENN-DDT strain, deltamethrin resistance is almost completely (98%) negated by the cytochrome P450 synergist piperonyl butoxide (PBO), suggesting that deltamethrin resistance in this strain is almost exclusively metabolically mediated. This is in contrast to permethrin resistance which is largely mediated by *kdr*, as PBO has a very limited synergistic effect on this phenotype (data not shown). The expression of deltamethrin resistance in SENN-DDT was less affected by multiple blood-feeding than that of DDT and permethrin resistance, while malathion resistance was largely unaffected. This is a confirmation of a previous study noting that blood does not have a strong effect on malathion toxicity [37]. These data suggest that multiple blood-feeding exerts a significant enough effect on the regulation of those detoxification enzymes also associated with insecticide resistance to maintain the expression of resistance in aging females, or at least to reduce the decline in resistance expression with age. Furthermore, the presence of *kdr* likely enhances this effect on the maintenance of resistance phenotype in blood-feeding females. This is suggested by the observation that the resistant phenotype produced by multiple blood meals is maintained for a prolonged period after feeding for DDT and permethrin, but the effect on deltamethrin resistance is restricted to the period immediately after feeding. This may be related to the presence of the blood-meal itself, rather than lasting metabolic changes induced by multiple feeding. However, it is more likely that variation in the relative contributions of metabolic detoxification and *kdr* to the expression of each phenotype account for these differences.

These data also suggest that *kdr* does not act independently of metabolic mechanisms in SENN-DDT. Although resistance to DDT is often primarily mediated by *kdr* in the closely related malaria vector *An. gambiae* (reviewed in [35]), the decreasing expression of the resistance phenotype with age in the control (non-blood fed) and single blood meal cohorts in this study suggests that without increased GST activity induced by blood meals *kdr* becomes less efficient with age. However, DDT resistance is never completely lost, in contrast to Cytochrome P450-mediated deltamethrin resistance, suggesting that *kdr* does play a significant role in production of the DDT resistance phenotype, but that this phenotype is primarily mediated by metabolic detoxification in SENN-DDT.

Of particular importance in these results is the data showing that older females that had taken multiple blood meals were not necessarily more susceptible to insecticides than younger females, which has implications for any control methods designed to target older females using lower doses of insecticide [22]. Vector control efficacy cannot be restored in insecticide resistant populations using this method if older, potentially infective females are as resistant to insecticides as their younger counterparts.

This study also demonstrates the variability of detoxification enzyme expression/production with age and that a continuous decline of enzyme activity with age is not the universal enzymatic behaviour. Multiple blood meals evidently suppress Cytochrome P450 activity, as also suggested previously by the down regulation of 22 P450 transcripts in blood fed *An. gambiae* females [38]. As cytochrome P450 activity induces oxidative stress [39, 40], this down regulation is likely a mechanism to reduce basal oxidative stress in order to cope with that induced by the blood meal [38]. Blood feeding, either as a single or multiple meals, sustains a reduction in P450 activity until 21 days. Multiple blood meals are also significantly associated with elevated GST activity throughout the female’s lifetime. Changes in esterase activity do not appear to correlate with any of the insecticide resistance phenotypes recorded in SENN-DDT. The increase in esterase activity in older females that had taken multiple meals may be due to reproductive functions in the female [41, 42].

The basis of reduced toxicity was investigated by Halliday and Feyereisen (1987), but no conclusion could be drawn about how the effect was mediated. This study may provide some insight into this mechanism. From the biochemical analysis, it is evident that multiple blood-meals result in sustained GST activity and suppressed cytochrome P450 activity. This is a confirmation of previous microarray studies that demonstrated the reduction of transcript abundance of various cytochrome P450s, but the increase of various GST transcripts and several antioxidant enzymes in response to blood-feeding in both laboratory [27] and field caught anophelines [38]. Muller *et al.* (2008) speculate that the response observed may be the mosquito’s system trying to shift towards a reduced state of oxidative stress as the activity of cytochrome P450s tend to induce oxidative stress [39, 40]. As reactive oxygen species concentration in the gut is relieved by a blood-meal [43], it is possible that the modulation of oxidative stress may play an important role in the effect of blood-feeding-induced reduction of insecticide toxicity and is a factor that is worth investigating.

*Anopheles gambiae* females generally forego sugar feeding if possible and feed frequently and preferentially on blood [10]. This may be true for *An. arabiensis* as well. The effect of multiple blood meals on the subsequent longevity of females is important. Mated *An. gambiae* females show reduced survivorship on sugar and blood than those that have not mated [44], suggesting a reproductive cost to these females. The present study mirrored this effect in insecticide susceptible SENN females in which multiple blood feeding increased the longevity of unmated, but not mated females. These data suggest that mated females shift their resources towards egg production at the expense of longevity. Interestingly, this effect was not mirrored in resistant SENN-DDT, where multiple blood feeding increased longevity regardless of mating status.

## Conclusions

In conclusion, multiple blood feeding is associated with the maintenance of DDT and pyrethroid resistance in aging *An. arabiensis* females harbouring *kdr* and metabolic resistance mechanisms. Previous studies have demonstrated a reduction in ROS after a blood-meal [43]. The effect observed in this study may be mediated by a reduction of oxidative stress in order to cope with ingested blood, which in turn reduces ROS-mediated insecticide toxicity. This effect is likely mediated by GSTs. The importance of GST-mediated defence against oxidative stress on pyrethroid toxicity has already been demonstrated [45]. Further investigations into these assertions are in progress (unpublished data). Multiple blood meals also favour survivorship of insecticide resistant females. Multiple blood-feeding is a common occurrence in anopheline mosquitoes. This does not only have reproductive and epidemiological consequences, but may also affect insecticide based malaria vector control measures directed against insecticide resistant vector populations.
